# Synthesis of Perovskite Nanocrystals and Their Photon-Emission Application in Conjunction With Liquid Crystals

**DOI:** 10.3389/fchem.2020.00574

**Published:** 2020-07-28

**Authors:** Weixi Lin, Qiumei Nie, Xiao-Fang Jiang, Xinshuai Jiang, Kai Wang, Lingling Shui, Shashank Priya, Guofu Zhou, Xiaowen Hu

**Affiliations:** ^1^Guangdong Provincial Key Laboratory of Optical Information Materials and Technology, Institute of Electronic Paper Displays, South China Academy of Advanced Optoelectronics, South China Normal University, Guangzhou, China; ^2^SCNU-TUE Joint Lab of Device Integrated Responsive Materials (DIRM), National Center for International Research on Green Optoelectronics, South China Normal University, Guangzhou, China; ^3^Material Research Institute, Pennsylvania State University, University Park, PA, United States; ^4^Academy of Shenzhen Guohua Optoelectronics, Shenzhen, China

**Keywords:** perovskite, nanocrystal, synthesis, display, liquid crystal

## Abstract

Perovskite nanocrystals have attracted worldwide attention due to their outstanding optical versatility, high photoluminescence quantum yields, and facile synthesis. In this review, we firstly revisit the synthetic methods for perovskite nanocrystals (PNCs), including hot injection, anion exchange, solvothermal reaction, etc. In the meantime, we discuss effects of the different synthetic methods on the properties of PNCs, including the crystal size, emission spectral feature, quantum yield, etc., followed by several optimizing strategies. Finally, lasing and display applications of these PNCs in combination with liquid crystal materials are discussed thoroughly. Outlooks on the challenges and opportunities of these nanocrystalline materials in terms of adjunct applications with liquid crystals have been presented at the end, which are highly promising for next-generation light emission applications.

## Introduction

The term “perovskite” comes from German mineralogist Gustav Rose, who stumbled upon the mineral CaTiO_3_ in The Urals of Russia and named it *perovskite* in honor of the Russian mineralogist Lev A. Perovski (Turkevych et al., 2017). Nowadays, perovskites refer not only to the minerals (Chen and Chen, 2019) but also to a material category having a similar crystallographic structure to that of CaTiO_3_, with increasing number of new components being added to the family. The general molecular formula of perovskites can be expressed as ABX_3_ (Li et al., 2008; Carpenter and Howard, 2009), where A is a monovalent cation that can either be an organic cation (such as CH_3_${\text{NH}}_{3}^{+}$, CHN_2_${\text{H}}_{4}^{+}$, etc.) or an inorganic cation (such as Cs^+^), B is an inorganic metal cation (such as Pb^2+^, Sn^2+^, etc.) with a smaller ion radius, and X is a halogen anion of Br, Cl, I, or their combination. In the perovskite lattice, the B-position cation is surrounded by six non-boring halogen atoms forming a BX_6_ octahedral structure which is mutually connected to form a three-dimensional (3D) network; the A-position cation is scattered in the void center of the BX_6_ 3D network to give a stable state (Yi et al., 2016). Based on the crystallographic A-site component, there are two sub-categories of perovskite: (i) hybrid organo-inorganic perovskites (HOIPs) (Zhang et al., 2015) that contain a small organic molecule cation at the A site and (ii) all inorganic perovskites (AIPs) (Protesescu et al., 2015) that contain an inorganic A-site cation. In HOIPs, the A-site cation is usually an amine molecule and has been investigated starting early 1978 (Weber, 1978) and has become a hot research topic in the field of photovoltaics in the past decades (Mitzi, 2000), leading to a quick increase of the solar–electricity power conversion efficiency (PCE) from 3 to 24% (Wang et al., 2019). In comparison, AIPs usually employ a cesium as the A-site cation and has a general composition of CsPbX_3_ (X=Cl, Br, I), which was discovered in 1958 (Chen and Chen, 2019). Compared to HOIPs, AIPs have higher thermal stability, but poorer α-phase stability. Nevertheless, there is a wider scope of researches focusing on the AIPs for optoelectronic applications (Green et al., 2014).

Fundamentally, most 3D perovskites have superior electronic band structures with a direct band gap of high tunability, long carrier diffusion length thanks to the polaron coupling effect, and other benign optoelectronic features (Stranks et al., 2015; Liang et al., 2019; Li et al., 2019). In parallel, the ultrahigh photoluminescence quantum yield (PLQY) of over 90% found in perovskites indicated a broad application prospect for this high-percentage radiative recombination. On the basis of the above intriguing features, nanostructured perovskite crystals are of great interest due to multiple quantum-confined effects. The most widely known perovskite nanocrystals are the perovskite quantum dots (PQDs) which have sizes in the nanometer scale that are less than or close to exciton Bohr radius, leading to a quasi-splitting electronic band structure (Li et al., 2019). Compared with the bulk perovskites, these perovskite nanocrystals (PNCs) have a distinct quantum-confined effect, leading to various physical and chemical insights. In terms of emission, PQDs have a higher color purity, wider color gamut, and lower cost processing compared to traditional quantum dots (QDs), leading to broader applications in display (Liu M. et al., 2017), lighting (Quan et al., 2018; Wei et al., 2019; Xuan et al., 2019), solar cells (Wang et al., 2019; Zhang et al., 2019), photodetectors (Miao and Zhang, 2019), and lasing (Jia et al., 2017; Evans et al., 2018).

Liquid crystals (LCs) represent a category of soft matter combining crystalline-like solid ordering with fluid-like behavior and were first recognized by Reinitzer (1888). What makes LCs unique is that they are fluids, yet exhibiting long-range order: either the orientation or position, or both, of the phase building blocks are correlated over a long distance (Lagerwall and Scalia, 2012). LCs include many phases, among which the most well known are nematic, smectic A, and chiral nematic (also known as cholesteric) phases. LCs cannot emit light, but it can selectively transmit or reflect light of specific wavelengths based on the internal arrangement of the LC molecules. For example, cholesteric liquid crystals (CLCs) have a unique periodic spiral structure. When CLCs present a planar arrangement structure, that is, the direction of the spiral axis of the crystals is perpendicular to the glass substrate, Bragg reflection will occur (Zola et al., 2019). Based on this optical property, some applications like liquid crystal display (Chen H. W. et al., 2018; Ko et al., 2018) and liquid crystal laser (Coles and Morris, 2010; Ortega et al., 2017; Chen L. J. et al., 2018) have been developed. As there is a growing number of researches on PQDs' display/lasering/lighting applications (Liang et al., 2019), here, we revisit the ongoing researches on PQD materials in terms of their chemistry in synthesis and applied physics in multiple emission applications in conjunction with LCs. Briefly, this review is composed of a first revisit of the synthetic methods for HOIP and AIP nanocrystals [including solvothermal synthesis, anion exchange, hot injection, ultrasonication, etc., followed by the doping and toxicity modification methods of perovskite nanocrystals (NCs)] and the prospective of a perovskite:liquid crystal composite for future display/lasering/lighting applications.

## Synthesis and Modification of HOIP and AIP Nanocrystals

So much effort has been devoted to developing reliable, simple but efficient strategies for preparing HOIP and AIP nanocrystals, and these approaches can be classified either as “top-down” or “bottom-up” (Shamsi et al., 2019). Top-down strategies comprise a fragmentation and structuring of macroscopic solids, either mechanically or chemically, whereas the bottom-up routes start with molecules and ions and proceed *via* gas- or liquid-phase chemical reactions. Here, we will focus on the liquid-phase methods of bottom-up strategies because it has been proven that these methods are the best for the fabrication of well-defined colloidal NCs among all the bottom-up approaches (de Weerd et al., 2018; Wang et al., 2018).

### Synthesis of HOIP Nanocrystals

**Figure 1:**
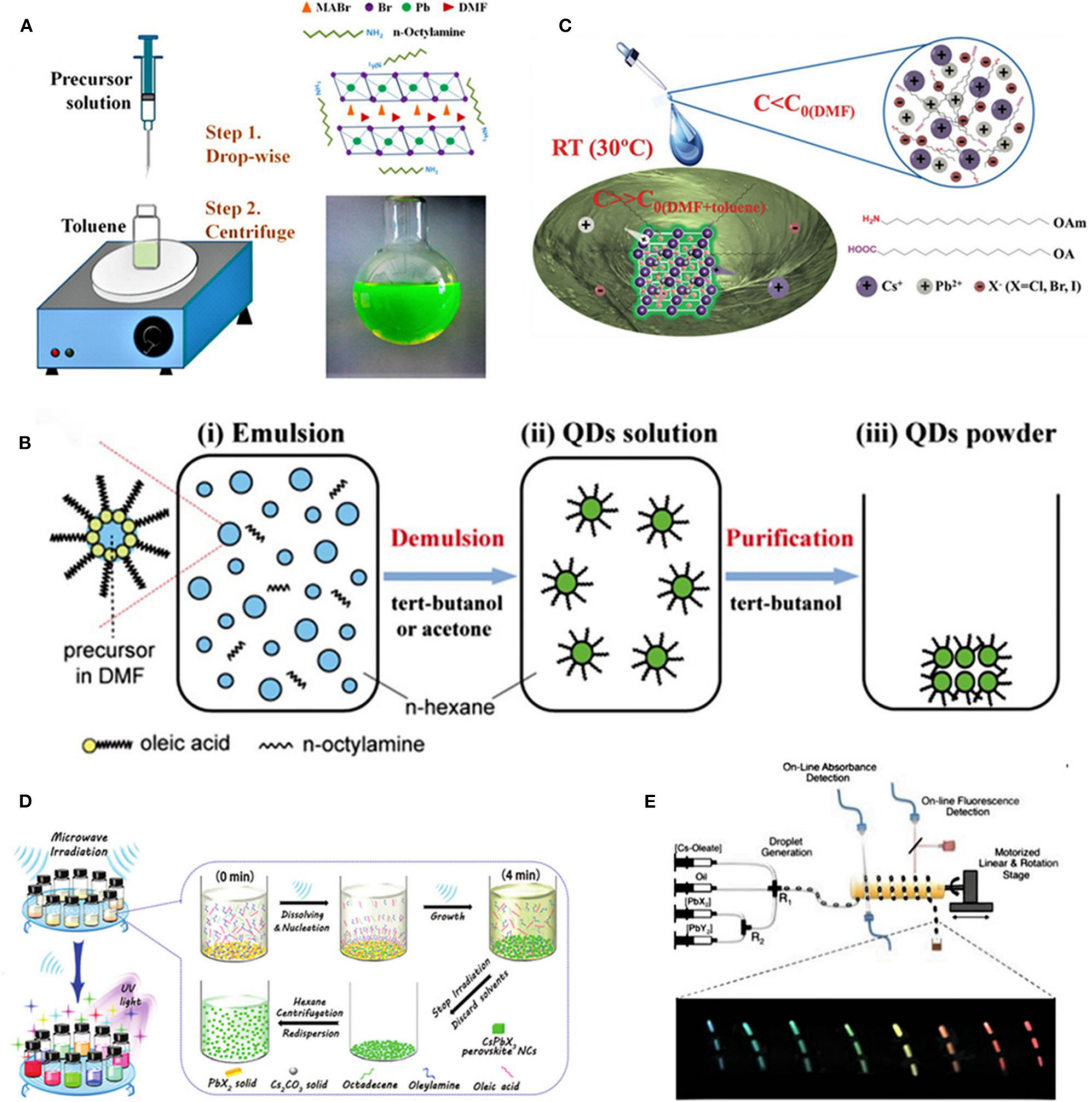
**(A)** Schematic illustration of the synthesis procedures for the ligand-assisted reprecipitation (LARP) process (Zhang et al., 2015). Reproduced with permission. Copyright 2015, American Chemical Society. **(B)** Emulsion synthesis (Huang et al., 2015). Reproduced with permission. Copyright 2015, American Chemical Society. **(C)** Supersaturated recrystallization (Li et al., 2016). Reproduced with permission. Copyright 2016, Wiley-VCH Verlag GmbH & Co. KGaA, Weinheim. **(D)** Microwave irradiation (Long et al., 2017). Reproduced with permission. Copyright 2017, Royal Society of Chemistry. **(E)** Microfluidic reaction (Lignos et al., 2016). Reproduced with permission. Copyright 2016, American Chemical Society.

A simple preparation method for PNCs (Schmidt et al., 2014) was firstly reported in 2014 by mixing MABr, PbBr_2_/*N, N*-dimethyl formamide (DMF) solution with octadecene (ODE), oleic acid (OA), and octadecyl ammonium bromide. The resultant cubic MAPbBr_3_ NCs displayed absorption and emission peaks respectively at 527 and 530 nm. The PLQY of the NCs was only 17% due to considerable auto-absorption, but the nanoparticles were proven to be kept stable in a solid state and maintained dispersed in aprotic, moderate-polarity organic solvents for more than 3 months. Subsequently, such approach has been developed into ligand-assisted reprecipitation (LARP) and emulsion synthesis. A LARP reaction can be achieved *via* simply pouring two precursor solutions into one reaction container to induce supersaturated precipitation at room temperature. Later, Zhang et al. (2015) firstly reported the general synthesis of MAPbBr_3_ QDs by dissolving a MAPbBr_3_ precursor consisting of PbBr_2_, MABr, *n*-octylamine (OAm), and OA into DMF to obtain a clear precursor solution, in which DMF acted as good solvent to dissolve the inorganic salts and small molecules, followed by dribbling the precursor solution into a vigorously stirred toluene. The long-chain ligands and acids used in the reaction played roles in controlling the crystallization process and stabilizing the formed colloidal QDs, and the supersaturation induced by the solubility change with solvent mixing contributed to the control of the crystallization process, which led to yellowish-green colloidal perovskite QDs with an average diameter of 3.3 nm and an enhanced PLQY up to 70% on account of the increase of the exciton binding energy due to the size reduction as well as proper chemical passivations of the Br-rich surface (Figure 1A). Similarly, perovskites with a tunable band gap, such as MAPbX_3_ (X = Cl, Cl/Br, Br, Br/I, I), can be prepared with a luminescence wavelength from 407 to 734 nm. Afterwards, Ling et al. (2016) prepared colloidal MAPbBr_3_ nanoplates (NPLs), which exhibited bright photoluminescence at 529 nm with a full width at half maximum (FWHM) of 20 nm and PLQYs up to 85%, *via* exempting amine halide by using extra organic solvents (such as OA and ODE). The PNCs prepared through this method possessed both an octylammonium bromide capping ligand and long-chain capping ligands, which led to a better moisture stability that stabilized in air with considerable humidity (~55%) for at least 1 week.

Emulsion synthesis mainly consists of two steps: emulsion formation and demulsification (Huang et al., 2015). The emulsion was obtained by mixing a pair of immiscible polar solvents (DMF and *n*-hexane), nonpolar solvents, and surfactants (OA) and then adding a demulsifier (such as *tert*-butanol, acetone, etc.) to initialize the mixing of precursors, which could induce solubility change and further drive the nucleation and crystallization of PQDs. As shown in Figure 1B, emulsion-synthesized HOIPs were prepared by dropping ABr-DMF and PbBr_2_-DMF into the mixed solution of hexane, OA, and *n*-octylamine, then the resultant monodisperse QDs were accomplished in a water-in-oil emulsion system and presented a tunable size from 2 to 8 nm as well as a satisfying PLQY in the range of 80–92%. The general crystallization principle of emulsion is similar to the LARP process; the only difference is how to realize a supersaturated environment. For LARP, the change of solubility induced by the mixing of solvents is a key step to driving the QD formation. For emulsion, forming a microreactor *via* the synthesis of the solvent is the prerequisite for crystallization.

Ultrasonication also contributes to preparing HOIPs. Jang et al. (2016) reported on the bulk MAPbX_3_ crystal synthesis *via* the ultrasonication method. They ground the mixing precursors of MABr and PbBr_2_ powders to disperse them in mixed toluene and OAm and obtained MAPbX_3_ NPLs by ultrasonic treatment. During the reaction process, OAm acted as a sufficiently protective ligand to stabilize the surface of the NCs and prohibit aggregation into a large particle, while toluene contributed to the precipitation of the products due to the higher solubilities of the two precursors in toluene. The solubility of the precursors in the solvent played an important role in the sonochemical synthesis of NCs, and ultrasonic irradiation favored dissolution and, hence, accelerated the reaction while resulting in the precipitation of products. The resultant NCs with different compositions all manifested uniform plate-type morphologies with an average size of 10 nm, and bright emission could be observed over a wide wavelength range of 400–800 nm accompanied by different band gaps (3.1–1.5 eV).

### Synthesis of AIP Nanocrystals

In the past few years, CsPbX_3_ is the most widely studied and used AIP. The facile colloidal synthesis of CsPbX_3_ PNCs with cubic shape and cubic crystal structure was firstly reported in 2015 with the hot injection method (Protesescu et al., 2015) consisting of two steps. Firstly, Cs_2_CO_3_ is added to a mixed solvent of ODE and OA under nitrogen to form a precursor solution at high temperature. Then, the precursor is dropped into a mixed solution of ODE, PbX_2_ (X = Cl, I, Br), OAm, and OA (150–200°C). After a few seconds of reaction, the mixture is quickly transferred to an ice bath to form the colloidal CsPbX_3_ AIP solution with the aid of the ionic nature of chemical bonding in the solution phase and fast nucleation/growth rates. Through compositional modulations and quantum size effects, the band gap energies and emission spectra could be easily tuned over the whole visible spectral region of 410–700 nm, while the size of the NCs could be tuned in the range of 4–15 nm owing to the exciton Bohr diameter of up to 12 nm; hence, the resultant NCs exhibited both compositional band gap engineering and size tunability of their band gap energies. The PLQYs of the AIP nanocrystals prepared by hot injection are as high as 50–90%, where the PLQYs of CsPbBr_3_ could even reach 95%. Similarly, FAPbX_3_ could also be prepared, and the as-synthesized HOIPs are able to achieve stable iodine-contained perovskite NCs, with efficient emissions in the red and near-IR regions. Soon afterwards, a more convenient and direct synthesis (Wang et al., 2015) of CsPbX_3_ with low threshold, wavelength-tunable, and ultrastable stimulated emission was achieved by modifying Protesescu's method.

The preparation of CsPbBr_3_ PNCs *via* supersaturated recrystallization at room temperature was firstly reported in 2016 (Li et al., 2016). Supersaturated recrystallization means that supersaturated ions will precipitate in the form of crystal when the constrainedly sustentative non-equilibrium state of a soluble system is activated by stirring or impurity. As presented in Figure 1C, CsX and PbX_2_ (X = Cl, Br, I, or their mixture) were chosen as ion sources and then dissolved in DMF, with OAm and OA as surface ligands. Li et al. (2016) calculated and designed a specific dosage so that the concentration of the ions was less than their solubility in the DMF. Subsequently, the solution obtained from the above reaction was added to an antisolvent such as toluene so that CsPbBr_3_ QDs could precipitate from the solution system. OAm and OA were used to control the size of the PNCs through surface functionalization to disperse them in the complex solvent system. Though crystallized at room temperature, the PQDs prepared by this method showed good photoluminescence (PL) performance, with high PLQYs of 70% for red (R), 95% for green (G), and 80% for blue (B) and good environmental stability that the retention rate could be 90% after aging 30 days in ambient conditions due to the enrichment of halogen ions found on the surface of PQDs, which resulted in self-passivation effect on defects. Moreover, the products formed the quantum well-like band alignment, improving the rate of radiative recombination. Later, Sun et al. (2016) prepared AIP nanocrystals with different nanostructures by using multiple ligands. They synthesized spherical, nanocubic, and rod-shaped CsPbBr_3_ NCs using surfactant ligands of *n*-caproic acid and octylamine, oleic acid and dodecane amine, and acetic acid and dodecane amine, respectively. Hence, by simply engineering the reaction system, multi-structured PNCs can be achieved.

Anion exchange is another common method for preparing AIP nanocrystals, particularly for tuning band gap through adjusting the halogen composition. Nedelcu et al. (2015) obtained full-spectra AIP nanocubes (edge lengths, 4–15 nm) with different anion components by anion exchange method, where they dissolved PbX_2_ into ODE and injected another perovskite toluene solution to easily realize the exchange due to the fast halide motion within the perovskite lattice and fast exchange dynamics of the halide ions in solution. Nedelcu and his coworkers effectively adjusted the fluorescence spectrum in the whole visible spectrum (410–700 nm) with the help of compositional modulations and quantum size effects and maintained high PLQYs of 20–80% with narrow emission line widths of 10–40 nm. Akkerman et al. (2015) also demonstrated the regulation of the optical properties by ion exchange. By mixing the synthesized PQDs with different lead halide salts, PQDs of any wavelength in the visible spectrum could be obtained after fast halogen ion exchange, with the crystal shape and morphology being unchanged. Such an anion exchange process does not change the structure and overall stability of the initial QDs. Moreover, fast ion exchange can also occur between perovskites with different halide ions, which may bring insights to innovate new complex material systems.

In 2016, Tong et al. reported on the preparation of AIP nanoplates *via* the ultrasonication method, which was versatile, polar solvent-free, and single-step (Tong et al., 2016). They firstly dissolved the corresponding precursor salts (Cs_2_CO_3_ and PbX_2_) into the ODE with OAm and OA as the surface ligands and then catalyzed the reaction by ultrasonication to initiate localized supersaturation and induce the formation of a cesium–oleate complex, which was soluble in nonpolar solvents. This leads to the cesium–oleate complex (soluble in a nonpolar solvent) further reacting with PbX_2_ in the presence of OAm and OA and directly converting into colloidal CsPbX_3_ in a one-step process. Furthermore, the thickness of the NPLs synthesized by ultrasonication was adjustable, with the average crystal sizes being in ranges of 10–15 nm and 8–12 nm for CsPbBr_3_ and CsPbI_3_, respectively, while the PLQY could still retain over 90%. Upscaling of the reactant amount does not change the optical properties of the resultant PNCs, indicating a highly practical method for massive production.

Apart from the ultrasonication, another reaction initiation method is the microwave irradiation. Long et al. (2017) reported on a high-throughput, single-step, rapid but controllable synthesis of multiple colloidal CsPbX_3_ NCs by microwave irradiation, as shown in Figure 1D. The heterogeneous solid–liquid mixture of ODE, OA, OAm, PbX_2_, and Cs_2_CO_3_ was added directly to a container in a microwave oven. After 4 min of static microwave irradiation, CsPbX_3_ NCs were synthesized with a yield of 40–60%. The obtained products were proven to be of cubic and rectangular shapes with size distributions from 10 to 13 nm and with PLQY ranging from 10.98 to 92.17%, and they covered a broad emission range from 410 to 692 nm. During the reaction process, the concentrations of the precursor ions in the liquid phase were quite low due to the low solubility of the solid precursors before microwave irradiation, then the precursors began to dissolve near the liquid–solid interface under microwave irradiation, followed by the nucleation of PNCs; finally, the reaction could be terminated simply through stopping the irradiation, which acted as thermal quenching. For this method, the reaction temperature could be up to the set point in a short time without a large temperature gradient. As a result the, preparation of AIP nanocrystals *via* microwave represents a simple but effective and controllable method.

Alternatively, other strategies have been applied to foster the synthesis of AIP nanoplates. Lignos et al. (2016) designed a rapid and mass transport controllable method to synthesize AIP quantum dots using a microfluidic reactor (Figure 1E). During the reaction process, the Cs oleate and PbX_2_ precursor are loaded into the precision syringe pump, and the precursor is transferred to the cross-junction agent through a fluorinated ethylene propylene tube. Finally, the mixture was heated to a predesigned temperature to realize the CsPbX_3_ QDs, which could cover an emission spectra range of 470–690 nm. Microfluidic reactors can precisely control key parameters such as the precursor type/concentration, fluid flow rate, and temperature/time, so the microfluidic reaction can be applied in a precisely controlled production.

Solvothermal synthesis is also a common synthesis method in recent years. In 2017, Chen et al. (2017) reported on a simple but efficient solvothermal synthesis for CsPbX_3_ PNCs. Cs_2_OAc and PbX_2_ were added to a stainless autoclave containing 1-ODE, OA, and OAm, which was then placed in a 160°C drum oven to execute the reaction. After the reaction, CsPbX_3_ nanocubes with PLQYs up to 80% covering the entire visible range and narrow emission line widths (from 12 to 36 nm) and CsPbX_3_ nanowires with a small diameter of 2.6 nm were both obtained, meaning that conversion between the different structures of CsPbX_3_ PNCs could be obtained through solvothermal synthesis. The control of the composition and structure is precise and the uniformity and crystallinity of the products are high.

Generally, LARP and emulsion synthesis are advantageous in room-temperature processing, single step, and short reaction time, which account for most current researched PNCs. However, due to the solvation and degradation of the resultant PNCs in polar solvents such as DMF, methanol, and ethanol, it is difficult to filtrate them from the reaction solution, which limits their applications in high-performance photoelectric devices. In contrast, hot injection secures high-quality PNCs, but suffers from low reaction yields. The anion exchange synthesis can effectively tune the optical properties of the NCs by anion exchange, while this will also introduce the defects and decrease the PLQY of the material. Alternatively, ultrasonication and solvothermal synthesis have been introduced, which unfortunately need longer reaction times and have difficulties in controlling the final crystal size. In contrast, the microfluidic method based on hot injection remains to be a promising technique, particularly when it can be coupled with both *in situ* emission and absorption tests immediately after the reaction, but one need to take consideration of the cost and investment on the setup.

### Modification of Perovskite NCs

The toxicity and instability of perovskite are inevitable issues in the synthesis of PNCs (Slavney et al., 2016). Most HOIPs and AIPs use PbX_2_ as one precursor, while Pb is highly toxic and harmful to the human body and not is environmentally friendly, making public acceptance difficult. Secondly, although colloidal perovskites prepared by different methods are stable in nonpolar solvents, when perovskite NCs are converted into a solid film for device application, their PLQYs drop significantly to 0.1%, owing to the loss of organic ligands that could result in NC aggregation and loss of quantum constraints. Here, we briefly discuss the proposed solutions to address the above issues.

To address toxicity issues, replacing Pb^2+^ or doping other metal cations in the B position to partially replace Pb^2+^ is the main research direction. Liu H. et al. (2017) reported on a method to partially replace Pb^2+^ with Mn to synthesize CsPb_*x*_Mn_1−−x_Cl_3_ QDs. They introduced the MnCl_2_ into the lead precursor and follow a hot-injection method to prepare the low-lead-content colloidal PQDs with a Mn substitution ratio up to 46%. By changing the ratios of Mn and Pb, QDs with different PL peaks could be obtained as well. Besides, upon Mn doping, the PLQYs of CsPbCl_3_ showed a decrease, with a maximum value of 54%. Chen L. J. et al. (2018) reported on the Sn-substituted perovskite of CsSnI_3_ PQDs *via* solvothermal synthesis. They chose SnI_2_ instead of PbI_2_ as the precursor to prepare AIP quantum dots which has a lower toxicity but good luminous performance. However, Sn^2+^ is easily oxidized into Sn^4+^ in the ambient atmosphere, so the synthesis process needs a high environmental requirement, which may increase the cost of the material.

Besides the toxicity concern, another big issue limiting the transition of perovskite technique is the instability of the material. To overcome this problem, imbedding the PNCs into an inert protective ligand has been researched. Xuan et al. (2018) reported on a method to prepare CsPbBr_3_:Cs_4_PbBr_6_ composite NCs by embedding CsPbBr_3_ into a new ligand of Cs_4_PbBr_6_. The composite NCs exhibited nearly monodisperse and regular hexagon shape; furthermore, the high-angle annular dark-field scanning transmission image and elemental mapping results exhibited that the Cs, Br, N, and Pb atoms were effectively and uniformly dispersed in the NCs, while the PLQYs of the NCs could reach as high as 83%. Alternatively, other matrix systems such as CsPbBr_3_ embedded into SiO_2_, SiO_2_/Al_2_O_3_, polymers are also attempted and show good luminescent performance. Among them, Cs_4_PbBr_6_ is an ideal choice because many results have shown that CsPbBr_3_ of cubic phase can match well with the specific lattice facet of Cs_4_PbBr_6_ and meanwhile passivate its surface defect to give a higher radiative recombination rate. The PLQYs of such NCs composites have been claimed to be 80% by Lou et al. (2019).

## PNCs: Liquid Crystals for Light Emission Applications

Many of the intriguing electronic and optical properties of PNCs, such as high optical absorption coefficient, direct energy band gap, large oscillator strength, long carrier lifetime, and high quantum efficiency, are particularly attractive for light-emitting devices. Compared with traditional organic semiconductors (like CdSe, ZnSe, PbS, etc.), one of the attractive features exhibited by PNCs is their functionality to tune their laser emission wavelengths over the entire visible range (390–790 nm) by exerting precise control over the synthesis conditions and compositional constitution. Meanwhile, the low-temperature solution processability of the PNCs permits the fabrication of devices in large scale under lower costs compared to the convectional fabrication methods for inorganic semiconductors, such as chemical vapor deposition (Dirin et al., 2016; Pandey et al., 2016; Huang et al., 2017).

### Lasing Application

Liquid crystals (LCs) are soft materials with several unique properties, such as high optical anisotropy, long-length-scaled softness and elasticity, and highly flexible manipulability by external sources (de Gennes and Prost, 1993). Among the different kinds of LCs, cholesteric LC (CLC) is particularly important given its twisting structure with high one-dimensional (1D) periodic modulation of the refractive index. As a 1D photonic crystal (Liu Y.-S. et al., 2017; Mani et al., 2017), CLCs have a photonic band gap (PBG), which can control the propagation of light of different wavelengths. By dispersing laser dyes (such as PNCs) into the LC matrix, or building a nanostructure composed of laser dyes and LCs, and meanwhile adjusting the PBG to match the PBG edge overlaps with the emission spectrum of the dyes to maximize the coupling effect, a dye-doped liquid crystal laser can be prepared (Coles and Morris, 2010). Such laser has the advantages of low threshold, excellent lasing properties, and no need of mirrors and other accessories. LC laser is a new emerging field of soft matter photonics which is expected to generate ultrathin and multifunctional laser source material systems. In parallel, PNCs, which can be used as laser dyes, are highly efficient in radiative transition with high PLQYs and can generate an amplified spontaneous emission (ASE), implying great potential in the application of light-emitting devices. Building novel material systems on the basis of both LCs and PNCs, light-emitting devices with high performance are expected.

**Figure 2:**
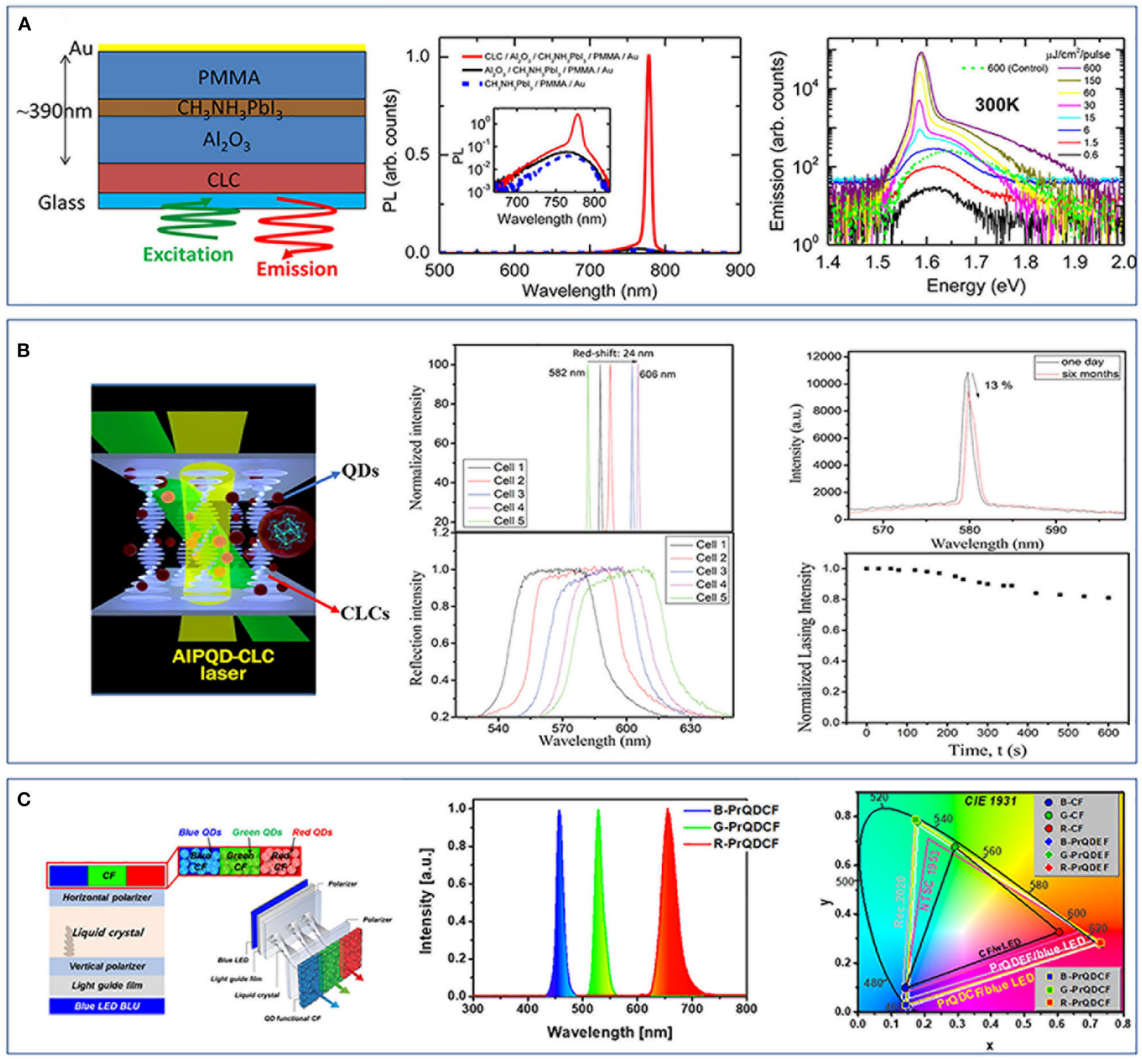
**(A)** Schematic illustration for a liquid crystal laser structure of a CH_3_NH_3_PbI_3_ film, a glass cholesteric liquid crystal (CLC) polymer reflector, an alumina substrate, and a metal backside reflector and its optical performance (Stranks et al., 2015). Reproduced with permission. Copyright 2015, American Chemical Society. **(B)** A liquid crystal laser doped with CsSnI_2_ quantum dots (QDs) and its optical performance (Chen L. J. et al., 2018). Reproduced with permission. Copyright 2018, American Chemical Society. **(C)** A vertically stacked liquid crystal display (LCD) structure of blue LED, light guide film, vertical polarizer, twisted nematic liquid crystals, horizontal polarizer, and QD functional color filters (CFs) and the optical performance of the CFs and LCD (Ko et al., 2018). Reproduced with permission. Copyright 2018, Springer Nature.

Stranks et al. (2015) reported on a method of preparing a high-performance structure composed of HOIP nanocrystals and CLCs. A HOIP of CH_3_NH_3_PbI_3_ film is sandwiched into a cavity between the alumina and polymer layer integrated in a glass CLC reflector (Figure 2A). With the aid of the Bragg reflection characteristics presented by CLCs and the amplification of PL caused by the band edge effect of the CLC-distributed feedback (DFB) optical resonator, ASE and lasing are successfully realized and enhanced. Moreover, the ASE threshold of the structured laser is two orders of magnitude lower than that of the laser without a CLC thin-film layer (Figure 2A). Chen L. J. et al. (2018) also dispersed CsSnI_3_ PQDs into CLCs and prepared a high-performance laser exhibiting a low threshold (150 nJ/pulse), narrow line width (0.20 nm), a wide tuning range of emission wavelengths (24 nm), and a good stability that the laser could retain approximately 87% of its initial lasing efficiency after half a year of storage under room temperature and high humidity of 60%, while the device still sustained a lasing efficiency higher than 80% of the initial value after the continuing excitation for 10 min (Figure 2B). The studies mentioned above show that the laser prepared by combining PNCs with CLCs has excellent performance and favorable stability.

### Display Application

Beyond the above laser applications, PNCs in liquid crystal display (LCD) also exhibit a big promise. Recently, quantum dot-enhanced films (QDEFs) have been introduced into LCD to work in collaboration with the light-emitting diode (LED) backlight units (BLUs) for minimizing the cross talks between the polarized emitting color of RGB (red, green, and blue) (Kim et al., 2013; Jiang et al., 2015). However, due to the loss of light in resin and traditional transparent film, the LCD with QDEF still has a large light power loss (Ko and Park, 2018). Ko et al. (2018) reported on an LCD that introduces the RGB-AIP QDs as functional color filters (CFs) within blue LED. The prepared LCD could eliminate the cross talks between RGB and greatly expand its color gamut (Figure 2C). The LCD had a vertically stacked structure of blue LED, light guide film, vertical polarizer, twisted nematic liquid crystals, horizontal polarizer, and QD functional CFs (Figure 2C). The specially designed ratio between the halogen elements of CsPbX_3_ made the luminescence peaks of the QDs match with the transmission wavelengths of the CFs, thus eliminating the cross talks between the BLUs. In addition, based on the excellent luminescence performance of perovskite QDs, an ultra-wide color gamut can be realized, and the improvement of this color gamut will help to reduce the pixels infinitely and generate ultra-high resolution.

## Conclusion and Outlook

We have briefly reviewed the synthetic methods for perovskite nanocrystals (PNCs) and discussed the effects of different synthetic techniques on the crystal properties. For the PNC materials, due to their high optical absorption coefficient, direct energy band gap, large oscillator strength, long carrier lifetime, and high quantum efficiency, light-emitting devices based on these PNCs are of particular interest. On the other hand, 1D cholesteric liquid crystals (CLCs) can manipulate the propagation of light, which has been well documented in related light-emitting and display applications. By taking advantage of both PNCs and CLCs, novel material systems incorporating both PNCs and LCs for multiple light-emitting applications are expected. So far, such a composite design of PNCs and LCs is starting to show its superior optoelectronic properties. A good example can be found in the PNC: LC composite for laser/displaying applications. Nevertheless, several material drawbacks of PNCs, such as their toxicity and instability issues, particularly at displaying applications which require highly optically/thermally stable materials, still remain to be addressed. In any case, as the researches on PNCs are still ongoing, and they will attract more attention in the future, we believe that the emerging PNC:LC composite holds great promise for advancing the development of novel light emission applications.

## Author Contributions

KW and XH proposed the manuscript review. WL and QN wrote the first draft. All authors contributed in the writing and review of the manuscript.

## Conflict of Interest

The authors declare that the research was conducted in the absence of any commercial or financial relationships that could be construed as a potential conflict of interest.

## References

[B1] Akkerman Q. A. D'Innocenzo V. Accornero S. Scarpellini A. Petrozza A. Prato M.. (2015). Tuning the optical properties of cesium lead halide perovskite nanocrystals by anion exchange reactions. J. Am. Chem. Soc 137, 10276–10281. 10.1021/jacs.5b05602 26214734 PMC4543997

[B2] Carpenter M. A. Howard C. J. (2009). Symmetry rules and strain/order-parameter relationships for coupling between octahedral tilting and cooperative Jahn–Teller transitions inABX_3_ perovskites. II. Application. Acta Crystallogr. Sect. B Struct. Sci. 65, 147–159. 10.1107/S0108768109000962 19299871

[B3] Chen D. Chen X. (2019). Luminescent perovskite quantum dots: synthesis, microstructures, optical properties and applications. J. Materials Chem. C 7, 1413–1446. 10.1039/C8TC05545A

[B4] Chen H. W. Lee J. H. Lin B. Y. Chen S. Wu S. T. (2018). Liquid crystal display and organic light-emitting diode display: present status and future perspectives. Light Sci. Appl. 7:17168. 10.1038/lsa.2017.168 30839536 PMC6060049

[B5] Chen L. J. Dai J. H. Lin J. D. Mo T. S. Lin H. P. Yeh H. C.. (2018). Wavelength-tunable and highly stable perovskite-quantum-dot-doped lasers with liquid crystal lasing cavities. ACS Appl. Mater. Interfaces 10, 33307–33315. 10.1021/acsami.8b08474 30198255

[B6] Chen M. Zou Y. Wu L. Pan Q. Yang D. Hu H. (2017). Solvothermal synthesis of high-quality all-inorganic cesium lead halide perovskite nanocrystals: from nanocube to ultrathin nanowire. Adv. Funct. Mater. 27:1701121 10.1002/adfm.201701121

[B7] Coles H. Morris S. (2010). Liquid-crystal lasers. Nat. Photonics 4, 676–685. 10.1038/nphoton.2010.184

[B8] de Gennes P. Prost J. (1993). The Physics of Liquid Crystals. New York, NY: Oxford University Press Inc.

[B9] de Weerd C. Gregorkiewicz T. Gomez L. (2018). All-inorganic perovskite nanocrystals: microscopy insights in structure and optical properties. Adv. Optical Mater. 6:289 10.1002/adom.201800289

[B10] Dirin D. N. Protesescu L. Trummer D. Kochetygov I. V. Yakunin S. Krumeich F.. (2016). Harnessing defect-tolerance at the nanoscale: highly luminescent lead halide perovskite nanocrystals in mesoporous silica matrixes. Nano Lett. 16, 5866–5874. 10.1021/acs.nanolett.6b02688 27550860 PMC5799875

[B11] Evans T. J. S. Schlaus A. Fu Y. Zhong X. Atallah T. L. Spencer M. S. (2018). Continuous-wave lasing in cesium lead bromide perovskite nanowires. Adv. Optical Mat. 6:982 10.1002/adom.201700982

[B12] Green M. A. Ho-Baillie A. Snaith H. J. (2014). The emergence of perovskite solar cells. Nat. Photonics 8, 506–514. 10.1038/nphoton.2014.134

[B13] Huang H. Bodnarchuk M. I. Kershaw S. V. Kovalenko M. V. Rogach A. L. (2017). Lead halide perovskite nanocrystals in the research spotlight: stability and defect tolerance. ACS Energy Lett. 2, 2071–2083. 10.1021/acsenergylett.7b00547 28920080 PMC5594444

[B14] Huang H. Zhao F. Liu L. Zhang F. Wu X.-G. Shi L.. (2015). Emulsion synthesis of size-tunable CH_3_NH_3_PbBr_3_ quantum dots: an alternative route toward efficient light-emitting diodes. ACS Appl. Mater. Interfaces 7, 28128–28133. 10.1021/acsami.5b10373 26652661

[B15] Jang D. M. Kim D. H. Park K. Park J. Lee J. W. Song J. K. (2016). Ultrasound synthesis of lead halide perovskite nanocrystals. J. Mater. Chem. C 4, 10625–10629. 10.1039/C6TC04213A

[B16] Jia Y. Kerner R. A. Grede A. J. Rand B. P. Giebink N. C. (2017). Continuous-wave lasing in an organic–inorganic lead halide perovskite semiconductor. Nat. Photonics 11, 784–788. 10.1038/s41566-017-0047-6

[B17] Jiang K. Sun S. Zhang L. Lu Y. Wu A. Cai C.. (2015). Red, green, and blue luminescence by carbon dots: full-color emission tuning and multicolor cellular imaging. Angew. Chem. Intern. Edit. 54, 5360–5363. 10.1002/anie.201501193 25832292

[B18] Kim T.-H. Jun S. Cho K.-S. Choi B. L. Jang E. (2013). Bright and stable quantum dots and their applications in full-color displays. MRS Bull. 38, 712–720. 10.1557/mrs.2013.184

[B19] Ko Y.-H. Jalalah M. Lee S.-J. Park J.-G. (2018). Super ultra-high resolution liquid-crystal-display using perovskite quantum-dot functional color-filters. Sci. Rep. 8:1. 10.1038/s41598-018-30742-w 30150618 PMC6110735

[B20] Ko Y.-H. Park J.-G. (2018). Novel quantum dot enhancement film with a super-wide color gamut for LCD displays. J. Kor. Phys. Soc. 72, 45–51. 10.3938/jkps.72.45

[B21] Lagerwall J. P. F. Scalia G. (2012). A new era for liquid crystal research: applications of liquid crystals in soft matter nano-, bio- and microtechnology. Curr. Appl. Phys. 12, 1387–1412. 10.1016/j.cap.2012.03.019

[B22] Li C. Lu X. Ding W. Feng L. Gao Y. Guo Z. (2008). Formability of ABX_3_ (X = F, Cl, Br, I) halide perovskites. Acta Crystallogr. B 64, 702–707. 10.1107/S0108768108032734 19029699

[B23] Li X. Wu Y. Zhang S. Cai B. Gu Y. Song J. (2016). CsPbX3Quantum dots for lighting and displays: room-temperature synthesis, photoluminescence superiorities, underlying origins and white light-emitting diodes. Adv. Funct. Mater 26, 2435–2445. 10.1002/adfm.201600109

[B24] Li Z. Chen Z. Yang Y. Xue Q. Yip H. L. Cao Y. (2019). Modulation of recombination zone position for quasi-two-dimensional blue perovskite light-emitting diodes with efficiency exceeding 5. Nat. Commun. 10:1027. 10.1038/s41467-019-09011-5 30833581 PMC6399279

[B25] Liang J. Chen D. Yao X. Zhang K. Qu F. Qin L.. (2019). Recent progress and development in inorganic halide perovskite quantum dots for photoelectrochemical applications. Small 16:e1903398. 10.1002/smll.201903398 31583803

[B26] Lignos I. Stavrakis S. Nedelcu G. Protesescu L. deMello A. J. Kovalenko M. V. (2016). Synthesis of cesium lead halide perovskite nanocrystals in a droplet-based microfluidic platform: fast parametric space mapping. Nano Lett. 16, 1869–1877. 10.1021/acs.nanolett.5b04981 26836149

[B27] Ling Y. Yuan Z. Tian Y. Wang X. Wang J. C. Xin Y.. (2016). Bright light-emitting diodes based on organometal halide perovskite nanoplatelets. Adv. Mater. 28, 305–311. 10.1002/adma.201503954 26572239

[B28] Liu H. Wu Z. Shao J. Yao D. Gao H. Liu Y.. (2017). CsPb_x_Mn_1−x_Cl_3_ perovskite quantum dots with high Mn substitution ratio. ACS Nano 11, 2239–2247. 10.1021/acsnano.6b08747 28145697

[B29] Liu M. Zhong G. Yin Y. Miao J. Li K. Wang C.. (2017). Aluminum-doped cesium lead bromide perovskite nanocrystals with stable blue photoluminescence used for display backlight. Adv Sci (Weinh) 4:1700335. 10.1002/advs.201700335 29201628 PMC5700652

[B30] Liu Y.-S. Lin H.-C. Yang K.-M. (2017). The opto-thermal effect on encapsulated cholesteric liquid crystals. Solid State Electron. 138, 89–93. 10.1016/j.sse.2017.09.005

[B31] Long Z. Ren H. Sun J. Ouyang J. Na N. (2017). High-throughput and tunable synthesis of colloidal CsPbX_3_ perovskite nanocrystals in a heterogeneous system by microwave irradiation. Chem. Commun. (Camb) 53, 9914–9917. 10.1039/C7CC04862A 28829077

[B32] Lou S. Zhou Z. Xuan T. Li H. Jiao J. Zhang H.. (2019). Chemical transformation of lead halide perovskite into insoluble, less cytotoxic, and brightly luminescent CsPbBr_3_/CsPb_2_Br_5_ composite nanocrystals for cell imaging. ACS Appl. Mater. Interfaces 11, 24241–24246. 10.1021/acsami.9b05484 31245989

[B33] Mani S. A. Amare J. R. Hadkar S. U. Mishra K. G. Pradhan M. S. Al-Johani H. (2017). Investigations of optical and thermal response of polymer dispersed binary liquid crystals. Mol. Cryst. Liquid Cryst. 646, 183–193. 10.1080/15421406.2017.1287478

[B34] Miao J. Zhang F. (2019). Recent progress on highly sensitive perovskite photodetectors. J. Mat. Chem. C 7, 1741–1791. 10.1039/C8TC06089D

[B35] Mitzi D. B. (2000). Organic–inorganic perovskites containing trivalent metal halide layers: the templating influence of the organic cation layer. Inorg. Chem. 39, 6107–6113. 10.1021/ic000794i 11151511

[B36] Nedelcu G. Protesescu L. Yakunin S. Bodnarchuk M. I. Grotevent M. J. Kovalenko M. V. (2015). Fast anion-exchange in highly luminescent nanocrystals of cesium lead halide perovskites (CsPbX_3_, X = Cl, Br, I). Nano Lett. 15, 5635–5640. 10.1021/acs.nanolett.5b02404 26207728 PMC4538456

[B37] Ortega J. Folcia C. L. Etxebarria J. (2017). Upgrading the performance of cholesteric liquid crystal lasers: improvement margins and limitations. Materials (Basel) 11:5. 10.3390/ma11010005 29267238 PMC5793503

[B38] Pandey M. Jacobsen K. W. Thygesen K. S. (2016). Band gap tuning and defect tolerance of atomically thin two-dimensional organic-inorganic halide perovskites. J. Phys. Chem. Lett 7, 4346–4352. 10.1021/acs.jpclett.6b01998 27758095

[B39] Protesescu L. Yakunin S. Bodnarchuk M. I. Krieg F. Caputo R. Hendon C. H.. (2015). Nanocrystals of cesium lead halide perovskites (CsPbX_3_, X = Cl, Br, and I): novel optoelectronic materials showing bright emission with wide color gamut. Nano Lett. 15, 3692–3696. 10.1021/nl5048779 25633588 PMC4462997

[B40] Quan L. N. Garcia de Arquer F. P. Sabatini R. P. Sargent E. H. (2018). Perovskites for light emission. Adv. Mater. 30:e1801996 10.1002/adma.201801996 30160805

[B41] Reinitzer F. (1888). Beiträge zur kenntniss des cholesterins. Monatsh. Chem. Verwandte Teile anderer Wissenschaften 9, 421–441. 10.1007/BF01516710

[B42] Schmidt L. C. Pertegás A. González-Carrero S. Malinkiewicz O. Agouram S. Mínguez Espallargas G.. (2014). Nontemplate synthesis of CH_3_NH_3_PbBr_3_ perovskite nanoparticles. J. Am. Chem. Soc 136, 850–853. 10.1021/ja4109209 24387158

[B43] Shamsi J. Urban A. S. Imran M. De Trizio L. Manna L. (2019). Metal halide perovskite nanocrystals: synthesis, post-synthesis modifications, and their optical properties. Chem. Rev. 119, 3296–3348. 10.1021/acs.chemrev.8b00644 30758194 PMC6418875

[B44] Slavney A. H. Smaha R. W. Smith I. C. Jaffe A. Umeyama D. Karunadasa H. I. (2016). Chemical approaches to addressing the instability and toxicity of lead–halide perovskite absorbers. Inorg. Chem. 56, 46–55. 10.1021/acs.inorgchem.6b01336 27494338

[B45] Stranks S. D. Wood S. M. Wojciechowski K. Deschler F. Saliba M. Khandelwal H.. (2015). Enhanced amplified spontaneous emission in perovskites using a flexible cholesteric liquid crystal reflector. Nano Lett. 15, 4935–4941. 10.1021/acs.nanolett.5b00678 25989354

[B46] Sun S. Yuan D. Xu Y. Wang A. Deng Z. (2016). Ligand-mediated synthesis of shape-controlled cesium lead halide perovskite nanocrystals via reprecipitation process at room temperature. ACS Nano 10, 3648–3657. 10.1021/acsnano.5b08193 26886173

[B47] Tong Y. Bladt E. Ayguler M. F. Manzi A. Milowska K. Z. Hintermayr V. A.. (2016). Highly Luminescent Cesium Lead Halide Perovskite Nanocrystals with Tunable Composition and Thickness by Ultrasonication. Angew Chem Int Ed Engl 55, 13887–13892. 10.1002/anie.201605909 27690323

[B48] Turkevych I. Kazaoui S. Ito E. Urano T. Yamada K. Tomiyasu H.. (2017). Photovoltaic rudorffites: lead-free silver bismuth halides alternative to hybrid lead halide perovskites. 10, 3754–3759. 10.1002/cssc.201700980 28660660

[B49] Wang N. Liu W. Zhang Q. (2018). Perovskite-based nanocrystals: synthesis and applications beyond solar cells. Small Methods 2:380 10.1002/smtd.201700380

[B50] Wang R. Mujahid M. Duan Y. Wang Z. K. Xue J. Yang Y. (2019). A review of perovskites solar cell stability. Adv. Funct. Mater 29:843 10.1002/adfm.201808843

[B51] Wang Y. Li X. Song J. Xiao L. Zeng H. Sun H. (2015). All-inorganic colloidal perovskite quantum dots: a new class of lasing materials with favorable characteristics. Adv. Mater. 27, 7101–7108. 10.1002/adma.201503573 26448638

[B52] Weber D. (1978). CH_3_NH_3_PbX_3_, ein Pb(II)-system mit kubischer perowskitstruktur / CH3NH3PbX3, a Pb(II)-**s**ystem with cubic perovskite structure. 33:1443 10.1515/znb-1978-1214

[B53] Wei Y. Cheng Z. Lin J. (2019). An overview on enhancing the stability of lead halide perovskite quantum dots and their applications in phosphor-converted LEDs. Chem. Soc. Rev. 48, 310–350. 10.1039/C8CS00740C 30465675

[B54] Xuan T. Huang J. Liu H. Lou S. Cao L. Gan W. (2019). Super-hydrophobic cesium lead halide perovskite quantum dot-polymer composites with high stability and luminescent efficiency for wide color gamut white light-emitting diodes. Chem.f Mater. 31, 1042–1047. 10.1021/acs.chemmater.8b04596

[B55] Xuan T. Lou S. Huang J. Cao L. Yang X. Li H.. (2018). Monodisperse and brightly luminescent CsPbBr_3_/Cs_4_PbBr_6_ perovskite composite nanocrystals. Nanoscale 10, 9840–9844. 10.1039/C8NR01266K 29785438

[B56] Yi C. Luo J. Meloni S. Boziki A. Ashari-Astani N. Grätzel C. (2016). Entropic stabilization of mixed A-cation ABX_3_ metal halide perovskites for high performance perovskite solar cells. Energy Environ. Sci. 9, 656–662. 10.1039/C5EE03255E

[B57] Zhang F. Zhong H. Chen C. Wu X.-g. Hu X. Huang H.. (2015). Brightly luminescent and color-tunable colloidal CH3NH3PbX3 (X = Br, I, Cl) quantum dots: potential alternatives for display technology. ACS Nano 9, 4533–4542. 10.1021/acsnano.5b01154 25824283

[B58] Zhang J. Hodes G. Jin Z. Liu S. (2019). All-Inorganic CsPbX_3_ perovskite solar cells: progress and prospects. Angew. Chem. Intern. Edition 58, 15596–15618. 10.1002/anie.201901081 30861267

[B59] Zola R. S. Bisoyi H. K. Wang H. Urbas A. M. Bunning T. J. Li Q. (2019). Dynamic control of light direction enabled by stimuli-responsive liquid crystal gratings. Adv. Mater. 31:e1806172. 10.1002/adma.201806172 30570775

